# The Role of Plant Origin Preparations and Phenological Stage in Anatomy Structure Changes in the Rhizogenesis of *Rosa* “Hurdal”

**DOI:** 10.3389/fpls.2021.696998

**Published:** 2021-09-07

**Authors:** Marta Joanna Monder, Paweł Kozakiewicz, Agnieszka Jankowska

**Affiliations:** ^1^Department of Dendrological Collections, Polish Academy of Sciences Botanical Garden—Center for Biological Diversity Conservation in Powsin, Warsaw, Poland; ^2^Department of Wood Science and Wood Preservation, Institute of Wood Sciences and Furniture, Warsaw University of Life Sciences—SGGW, Warsaw, Poland

**Keywords:** adventitious roots, biostimulant, root enhancer, tracheids, vessel, xylem

## Abstract

Most old roses are difficult to root when propagated by cuttings. This research focused on the response of stem cuttings of *Rosa* “Hurdal” to plant origin preparations used as rhizogenesis enhancers through changes to the anatomical structure of the basal part of the stem. Cuttings derived from shoots in four phenological stages were prepared for the experiment: flower buds closed (H1); fully flowering (H2); immediately after petals have fallen (H3); 7–14 days after petals have fallen (H4). The cuttings were treated with 0.4% indole butyric acid (IBA; Ukorzeniacz A_aqua_) or 0.2% naphthalene acetic acid (NAA; Ukorzeniacz B_aqua_), and with plant origin preparations: Algae extract (Bio Rhizotonic), Organic preparation (Root Juice^TM^), and Plant extract (Bio Roots). A high rooting percentage in comparison to the control (27.5%) was obtained after treatments of the H1 cuttings with Algae extract (90%), Organic preparation (80%), and Plant extract (75%). The H4 cuttings did not root, probably as a result of an overgrowing callus and limited xylem formation. The anatomical structure of the shoot differed in subsequent phenological stages during the period of rooting in various ways, depending on the rooting enhancer used for treatment. Numerous correlations between rooting percentage and anatomical structure were proved, including the key role of vascular bundles in increasing rooting percentage by widening the vessel diameter.

## Introduction

**Figure 1:**
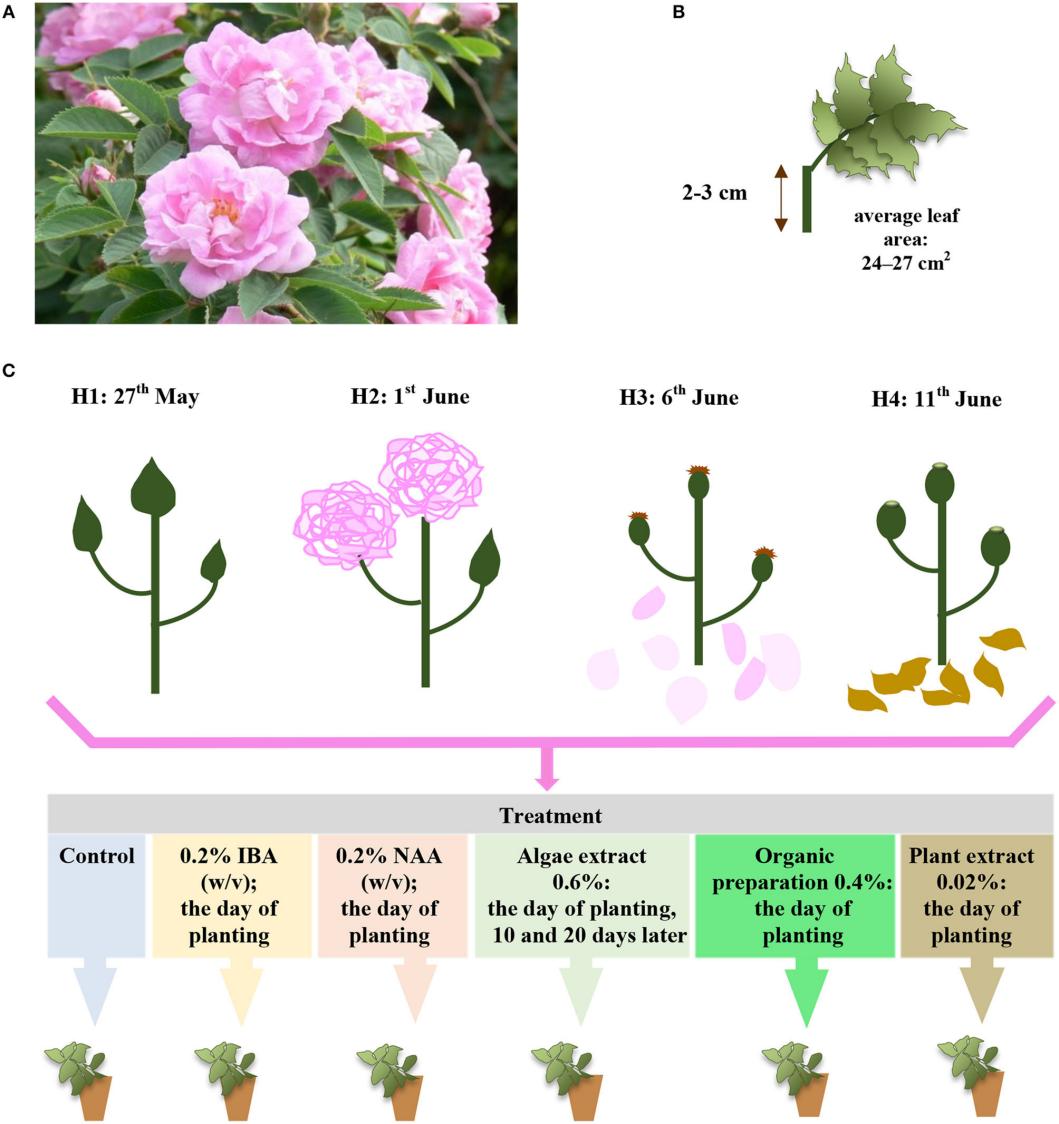
The *Rosa* “Hurdal” **(A)** single-node leaf cutting, **(B)** phenological stages of shoots in terms and their treatment in experiment **(C)** phenological stages in BBCH scale (Meier et al., 2009): H1−54,504 (flower buds still closed); H2−69,605 (full flowering; at least 50% of flowers open); H3−69,629 (end of flowering of the second-order stem—all petals have fallen); H4−70,701 [fruit set: beginning of hip (berry) growth on the main stem].

Roses belong to the oldest and mostimportant cultivated ornamental plants (Smulders et al., 2019), and have been significant in many fields of human life for thousands of years (Gustavsson, 1999). The majority of once-blooming old roses are valued for their winter hardiness (Gustavsson, 1999; Monder, 2014) and high resistance to pests and disease. The majority of old roses can also be successfully cultivated in the temperate zone in a climate with harsher winters on their own roots without budding on the rootstock (Gustavsson, 1999). Their maintenance in cultivation is important for biodiversity conservation and the preservation of the heritage of garden plants, and so for the implementation of the provisions of the Convention on Biological Diversity (CBD) made in Rio de Janeiro on 5 June 1992. Although old roses are commonly cultivated, they should, nevertheless, be used more often to revitalize historical properties and urban greeneries (Gustavsson, 1999; Monder, 2021). “Hurdal” is a valuable old rose and, originally, an Alba or Villosa Hybrid. This cultivar has been more widely known since it was brought to Norway in the second half of the nineteenth century. The shrubs are ca 3-m high, with long, almost thornless stems, and big grayish leaves. The flowers are deep pink, with 15–25 petals and a mild fragrance (Figure 1A). The hips are oval, ca 2-cm diameter, and red-orange. The shrubs exhibit great frost hardiness and are appropriate for cultivation in a hard Scandian climate (Gustavsson, 1999).

The use of single-node leafy stem cuttings is easy and economical, and it is the most common method of rose propagation used to grow shrubs on their own roots. However, the forming of adventitious roots in the cuttings of vascular plants is a very complicated process connected with exogenous and endogenous factors (Hartmann et al., 2011), whereby rhizogenezis in cuttings of wild (Hoşafçi et al., 2005) and old roses is believed to be especially difficult (Hoşafçi et al., 2005; Ginova et al., 2012), time-consuming, and often resulting in failure (Ginova et al., 2012; Monder et al., 2016). The process of rhizogenezis, prolonged to 12 weeks, exposes the cuttings to many stress factors, and their success is also strictly connected with the content of bioactive components in stock plants (Monder et al., 2016). The anatomical structure of the shoot (Amissah et al., 2008; Bryant and Trueman, 2015) is a crucial endogenous factor in adventitious root formation for the majority of vascular plants, including roses (Monder et al., 2017).

The growth and the development of plants in the growing season are connected with many changes in their biology. Phenology is a visible effect and a source of knowledge of periodic changes in plants (Meier et al., 2009) affected by additional environmental factors (Zheng et al., 2016), including roses (Monder, 2014, 2021). The crucial role of the phenological stage of shoots in roses harvested for the preparation of cuttings has been shown a few times (Pihlajaniemi et al., 2005; Monder et al., 2016, 2019; Monder and Pacholczak, 2018). The flowering time in once-blooming roses is followed by changes in carbohydrates, soluble proteins, chlorophyll a/b, and carotenoids content (Monder et al., 2016) as well as changes in the anatomical structure (Monder et al., 2017).

Various rooting enhancers, including plant hormones (Pihlajaniemi et al., 2005; Ginova et al., 2012) and biostimulants (Monder and Pacholczak, 2019; Monder et al., 2020) were used to encourage the natural ability of cuttings to undergo rhizogenesis and also to improve the quality and physiological parameters of rooted cuttings. Some of the preparations important in nursery production contained indole butyric acid (IBA) or naphthalene acetic acid (NAA) as standard rooting enhancers in the procedure of rooting cuttings (Hartmann et al., 2011). Previous research has proven the influence of a few substances used as rooting enhancers on anatomical structure, e.g., benzyladenine and naphthalene-1-acetic acid in Fuchsias (Wróblewska, 2013), Dogwood (Pacholczak et al., 2012), and Euphorbia hybrids (Fascella and Zizzo, 2009) or carbon dioxide (Costa et al., 2007), IBA, and NAA in roses (Dawa et al., 2018; Monder et al., 2019). However, the abovementioned research did not establish which changes in anatomy had an influence on the effectiveness of the process of rhizogenesis.

Synthetic chemicals used for plant production, protection, and cultivation are often replaced, or their usage is reduced, by preparations of natural origin that includes humic acids, seaweed, and Plant extracts. Their use provides a number of benefits in stimulating growth and protecting against biotic and abiotic stresses (Oosten et al., 2017). Preparations of natural origin are preferred in sustainable eco-friendly agriculture and recommended by the Official Journal of the European Union (OJEU, 2009a,b), the National Organic Program USDA (USDA, 2017), and the Organic Materials Review Institute (OMRI, 2021). The effect of their use depends on their composition and, especially, on phytohormones (Oosten et al., 2017). Three commercial plant origin preparations recommended by their manufacturers for rooting and replanting were used in the present research and named for the purposes of this work: Bio Rhizotonic—Algae extract (Canna Continental, Los Angeles, USA, 2021), Root Juice™–Organic preparation (BioBizz Worldwide B.V., Groningen, The Netherlands, 2017), and Bio Roots—Plant extract (General Hydroponics Europe/T.A. Terra Aquatica, Fleurance, France). More detailed information on these preparations is presented in Table 1. These preparations were produced with Plant extract as their base and contain many biologically active substances. The content of humic substances could lead to higher nutrient content in the tissues of the cuttings as well as positive metabolic changes (Nardi et al., 2015). The above preparations affected the content of chlorophyll a, b, carotenoids, soluble proteins (Monder et al., 2020), and carbohydrates in roses (Monder and Pacholczak, 2018) and can indirectly influence the effectiveness of rhizogenesis.

**Table 1:** Rooting enhancers used in the rooting of *Rosa* “Hurdal.”

**Name in work**	**Preparation and producer**	**Characteristic**	**Certificate**
IBA	Ukorzeniacz A_aqua_ (Himal, Poland)	0.4% IBA (indolebutyricacid)	
NAA	Ukorzeniacz B_aqua_ (Himal, Poland)	0.2% NAA (naphthaleneaceticacid)	
Algae extract	Bio Rhizotonic (Canna Continental, Los Angeles, USA, 2021)	Seaweed-based, 100% organic, N 0.6%, P 0.2%, K 0.6% vitamins such as B_1_, B_2_, and other biologically active substances	Organic Materials Review Institute (OMRI)
Organic preparation	Root Juice^TM^ (BioBizz Worldwide B.V., Groningen, The Netherlands, 2017)	Combination of humic acid and seaweed extracts, N 0.1%, P_2_O_5_ 0.1%, K_2_O 0.1%, Mg 0.03%, Fe 0.013%, Mn 0.002%, Zn 0.004%, B 0.025%, Cu 0.001%	National Organic Program (NOP); Control Union Certified EU; Good Soil Quality Mark; Point Vert; Organic Materials Review Institute (OMRI), Clean Green Certified
Plant extract	Bio Roots (GHE, France/T.A. Terra Aquatica, 2021)	Amino acids and oligosaccharins, fruit oil up to 1%; humic acids 1%; pectinate 1%; sodium alginate 3%; seaweed species 10%, organic matter 84%	European regulation EC No 834/2007 on organic agriculture (Certificaat Bio Roots, 2014)

The aims of this research were to recognize the response of cuttings *Rosa* “Hurdal” in anatomical structure to the plant origin preparations during the process of rhizogenesis. Moreover, we hypothesized that the response of cuttings prepared from shoots in four phenological stages may be different. Consistently, the various anatomical changes occurring in stock plants during the flowering period can affect rooting effectiveness and the quality of rooted cuttings. Analysis of changes in anatomical structure before and after the rooting process could reveal the reason for difficulties in the rooting of some rose cuttings.

## Materials and Methods

### Plant Material and Experimental Conditions

The plant material of *R*. “Hurdal” (Germany–Norway; origin unknown, *Rosa* × *alba* × *Rosa villosa*, Figure 1A) was obtained from shrubs growing in the National Collection of Rose Cultivars in the Polish Academy of Sciences Botanical Garden Center for Biological Diversity Conservation in Powsin, Warsaw, Poland.

The single-node leafy semi-hardwood shoot cuttings of *Rosa* “Hurdals” (Gustavsson, 1999) with an original leaf (leaf area 24–27 cm^2^) (Figure 1B) were taken from the middle part of flowering shoots (three to five buds) in four phenological stages (Figure 1C) according to BBCH scale in roses (Meier et al., 2009). In the present study, the letter H, accompanied by a subsequent number, was used to designate the phenological stage of shoots harvested for the experiment in four periods: H1—May 27, 54 504 (flower buds still closed), H2—June 1, 69 605 (full flowering; at least 50% of flowers open), H3—June 6, 69 629 (end of flowering of the second-order stem: all petals fallen), H4—June 11, 70 701 (fruit set: beginning of hip growth on the main stem) (Figure 1C). The shoots all had similar diameters, ranging from 5 to 6 mm.

The cuttings were inserted into multipot trays (6.6 × 6.6 cm) in peat (Karaska, Poland) sand (Vistula river) medium (mixture v:v 1:1; pH 6–6.5). The cuttings were treated with 0.4% IBA (Ukorzeniacz A_aqua_) or 0.2% NAA (Ukorzeniacz B_aqua_), and with plant origin preparations: Algae extract (Bio Rhizotonic), Organic preparation (Root Juice^TM^), and Plant extract (Bio Roots). The treatment and watering process is presented in detail in Figure 1C. Before planting, the basal parts (1 cm) of the stem were dipped in commercial rooting powder, containing 0.4% IBA or 0.2% NAA. The remaining cuttings were watered with plant origin preparations or, in the case of the control, water. The watering was performed with 10 cm^3^ per pot (98 cm^3^) according to the schema of the experiment. During the 10th and 20th days of rooting, the cuttings were watered with 10 cm^3^ water or Algae extract per pot (98 cm^3^) (Figure 1C). The rooting enhancers used in the experiment were purchased from companies in the sector (Table 1).

The experiment on rooting cuttings was conducted in the commercial nursery of M&M Kryt in Wola Prażmowska (51.56°N, 20.28°E), Poland. The rose cuttings were rooted in a foil tunnel with an electronically controlled misting system that maintained optimal climatic conditions (air temperature, 23–25°C; ambient relative humidity, 80–90%). The shading material limited the photosynthetic photon flux density to 130 μmol·m^−2^·s^−1^. To prevent fungal diseases, the following fungicides were applied to the cuttings regularly every 7–9 days starting from the day of planting: Previcur Energy 840 SL (propamocarb, 47.28%; fosetyl, 27.65%; Bayer, Poland), Amistar^®^ 250 SC (azoxystrobin, 250 g·dm^−3^; Syngenta, Poland), and Score 250 SC (difenoconazole, 250 g·dm^−3^; Syngenta, Poland).

### The Data Set of the Growth Parameters of the Cuttings

After 12 weeks, in the first days of August, the cuttings were dug out and cleaned from the medium. The rooted cuttings were earmarked for further evaluation.

The rooting percentage and the percentage of cuttings with a callus only were calculated (%) in relation to the number of planted cuttings.

The fresh weight (g) of the root system and the aboveground part were estimated for rooted cuttings, using an analytical balance (PS 6000/C/2, RADWAG, Poland). The root length (cm) was measured with a caliper from the origin of the root to the apical meristem of the primary and longest root.

### Anatomical Evaluation

Samples of shoots from cuttings were retrieved for anatomical research two times for every treatment in each phenological stage: before being planted in the rooting medium and on the 25^th^ day of the rooting process. Moreover, the samples were harvested and observed separately after 12 weeks of rhizogenesis. The shoot fragments were protected and stabilized in a mixture of anhydrous glycerine and 96% ethyl alcohol (v/v 1:1) in PAS Botanical Garden CBDC in Powsin, Warsaw, Poland. Slides were cut with a sledge microtome (pfm SLIDE 4003E, pfm medical AG, Germany) into slices 15- to 30 μm thick with cross- and longitudinal sections from the basal parts of shoots, in which rhizogenesis would have taken place. The scraps were cross-dyed with a solution of safranine. The studies were carried out using the light Olympus BX41 microscope (Olympus America Inc., New York, USA) with the Olympus DP25 digital camera connected to a computer with specialist Cell^*^B software (The Institute of Wood Sciences and Furniture, Warsaw University of Life Sciences—WULS, Warsaw, Poland) and an Olympus Vanox AHBT3 (Olympus America Inc., New York, USA) microscope. The measurements of cells and tissue elements were conducted with the specialist Cell^*^B software and cellsSens Standard 1.7.1. software in PAS Botanical Garden CBDC in Powsin, Warsaw, Poland. The width of the xylem, phloem, pith rays, and cortex, and, additionally, the early- and late-wood vessel diameter, were measured before and after 25 days of rooting cuttings and, in the shoots of the stock plant, cut on 5 August. The width of tissues between xylem and cortex was noted and observed after 25 days of rooting cuttings. All the observations were carried out on the basal part of the cuttings: 2–2.5 cm below buds in shoots.

### Statistical Analysis

The experiments were constructed as randomized complete block design (RCBD) for each maturity phase and involved 24 combinations altogether for two variables (treatment, phenological stage). Each combination of treatment in four phenological stages of shoots (Figure 1) included four replicas of 10 cuttings. A total of 960 cuttings were planted. Additional 10 cuttings earmarked for anatomical research were planted in each combination. Moreover, the anatomical research data were collected for each phenology stage of shoots before rooting them, also from the 10 cuttings.

The values in percentage were transformed by using the function ARCSIN(*x*)^1/2^ according to Bliss or *y* = *x*^2^ + (*x*^2^ + 1)^2^ to compare the means and carry out analyses. The data of anatomical parameters were analyzed by using two-factorial analysis of variance (ANOVA), and Duncan's honest significant difference test was used to determine the significance of differences between the means (α = 0.05) for two variables—the phenological stage and the rooting enhancer. For data of growth parameters, two-way analysis of variance (ANOVA) and Tukey's HSD were used (α = 0.05) for the same variables, respectively. Correlations between the data parameters of growth and anatomical structure in cuttings were carried out for each phenological phase and treatment together and separately.

The STATISTICA 10 software package (StatsoftPolska, Cracow, Poland) was used for statistical analysis.

## Results

### The Anatomy of the Shoots of *Rosa* “Hurdal” Stock Plants Changes Across the Different Phenological Stages That Cuttings Were Prepared in

**Figure 2:**
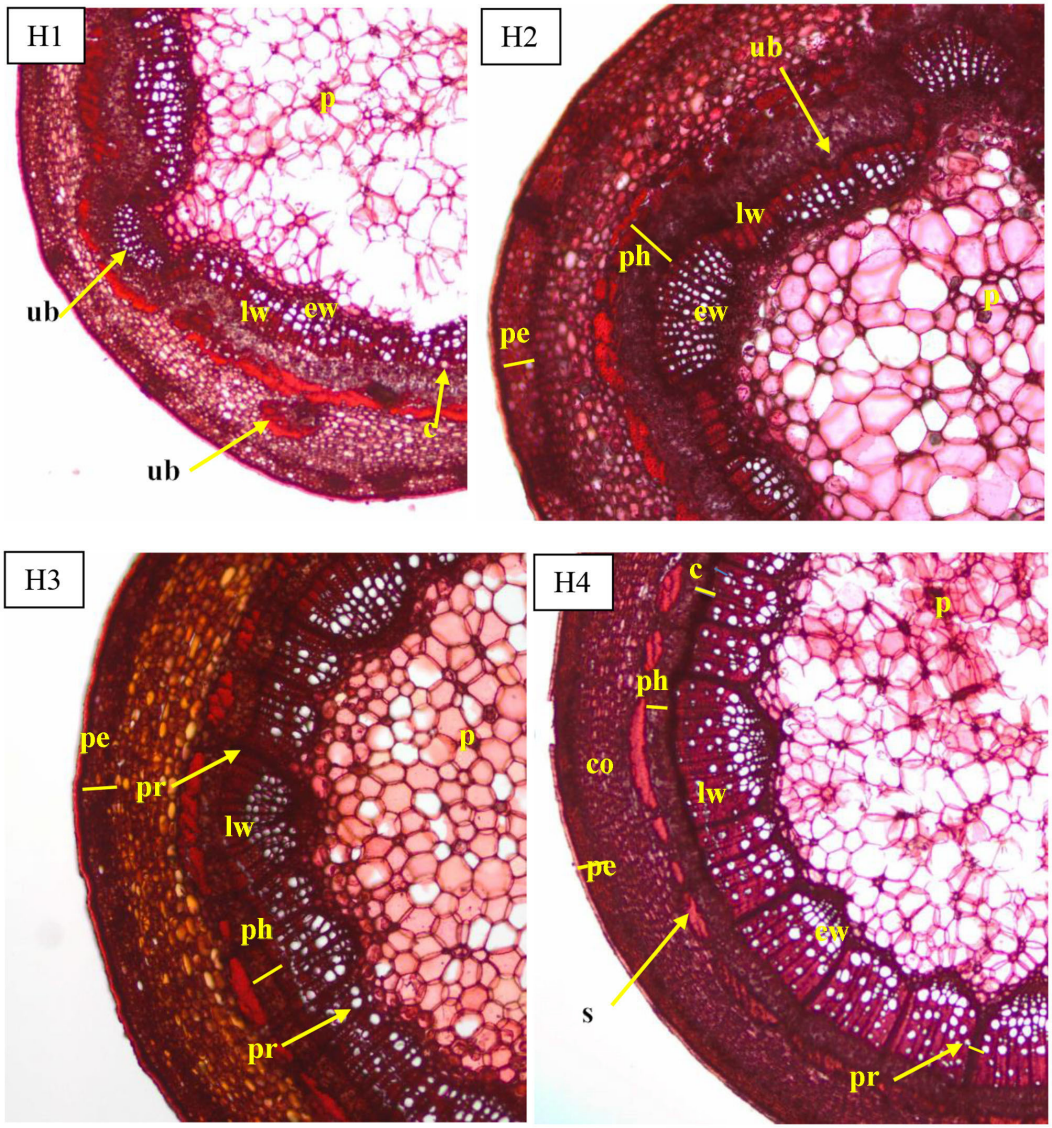
Transverse sections of the shoots before rooting in *Rosa* “Hurdal” in four phenological stages in BBCH scale (Meier et al., 2009): H1−54,504 (flower buds still closed); H2−69,605 (full flowering; at least 50% of flowers open); H3−69,629 (end of flowering of the second-order stem—all petals have fallen); H4−70,701 [fruit set: beginning of hip (berry) growth on the main stem]. Designation of signs: ub, unregular vascular bundles; c, cambium; co, cortex; pe, periderm; ph, phloem; p, pith; pr, pith rays; s, sclerenchyma; ew, earlywood; lw, latewood.

**Figure 3:**
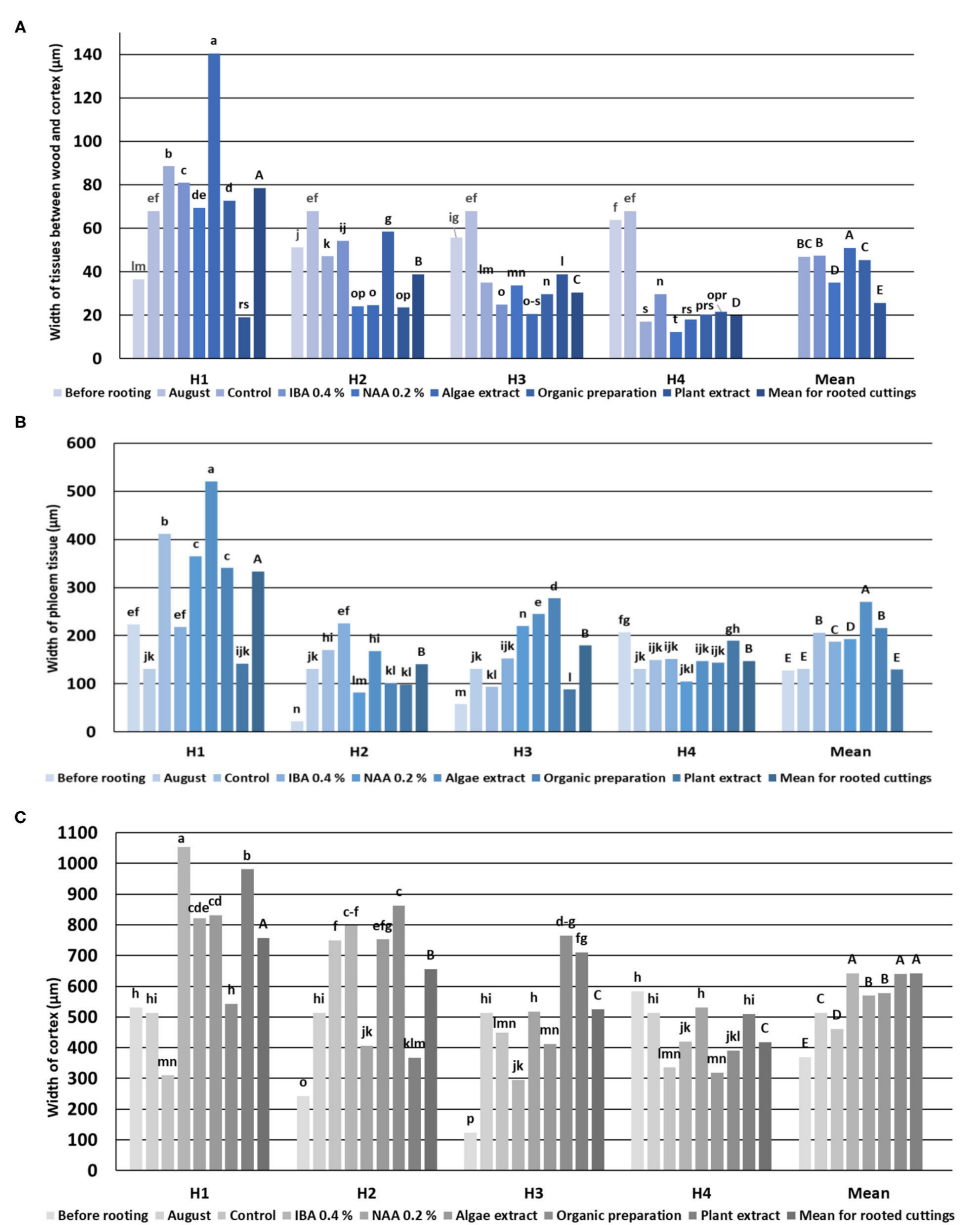
Width of *Rosa* “Hurdal” tissues between wood and cortex **(A)**, phloem **(B)**, and cortex tissue **(C)** in stems before rooting; cuttings after 24 days of the rhizogenesis process; and stems of stock plants cut in first days of August. Control—control cuttings without rooting enhancers; IBA 0.4%—Ukorzeniacz A_aqua_ (0.4% IBA); NAA, 0.2%—Ukorzeniacz B_aqua_ (0.2% NAA); Algae extract—watering with 0.6% Bio Rhizotonic (10 ml) after planting and 10 and 20 days later; Organic preparation—watering with 0.4% Root Juice™ (10 ml) after planting; Plant extract—watering with 0.02% Bio Roots (10 ml) after planting. H1−54 504 (flower buds still closed); H2−69 605 (full flowering; at least 50% of flowers open); H3−69 629 (end of flowering of the second-order stem—all petals have fallen); H4−70 701 [fruit set: beginning of hip (berry) growth on the main stem]. Different small letters indicate significant interactions between the phenological stage and treatment (two-way ANOVA). Different capital letters indicate significant differences between phenological stages. Duncan's test (α = 0.05) was used.

The periderm consisted in (i) a cork cambium with undifferentiated cells (phellogen), (ii) an outer layer (epidermis) with cells in regular rows and thick walls, and (iii) living cells with thickening walls in an inner layer arranged in three to four rows. The cortical parenchyma had a similar width to the vascular bundle ring visible on cross sections of all four phenological stages of shoots. The parenchyma consisted of large cells, uneven in size, and tightly arranged along the circumference of the shoot (Figure 2). The width of the cortex decreased from H1 to H4 (Figure 3).

**Figure 4:**
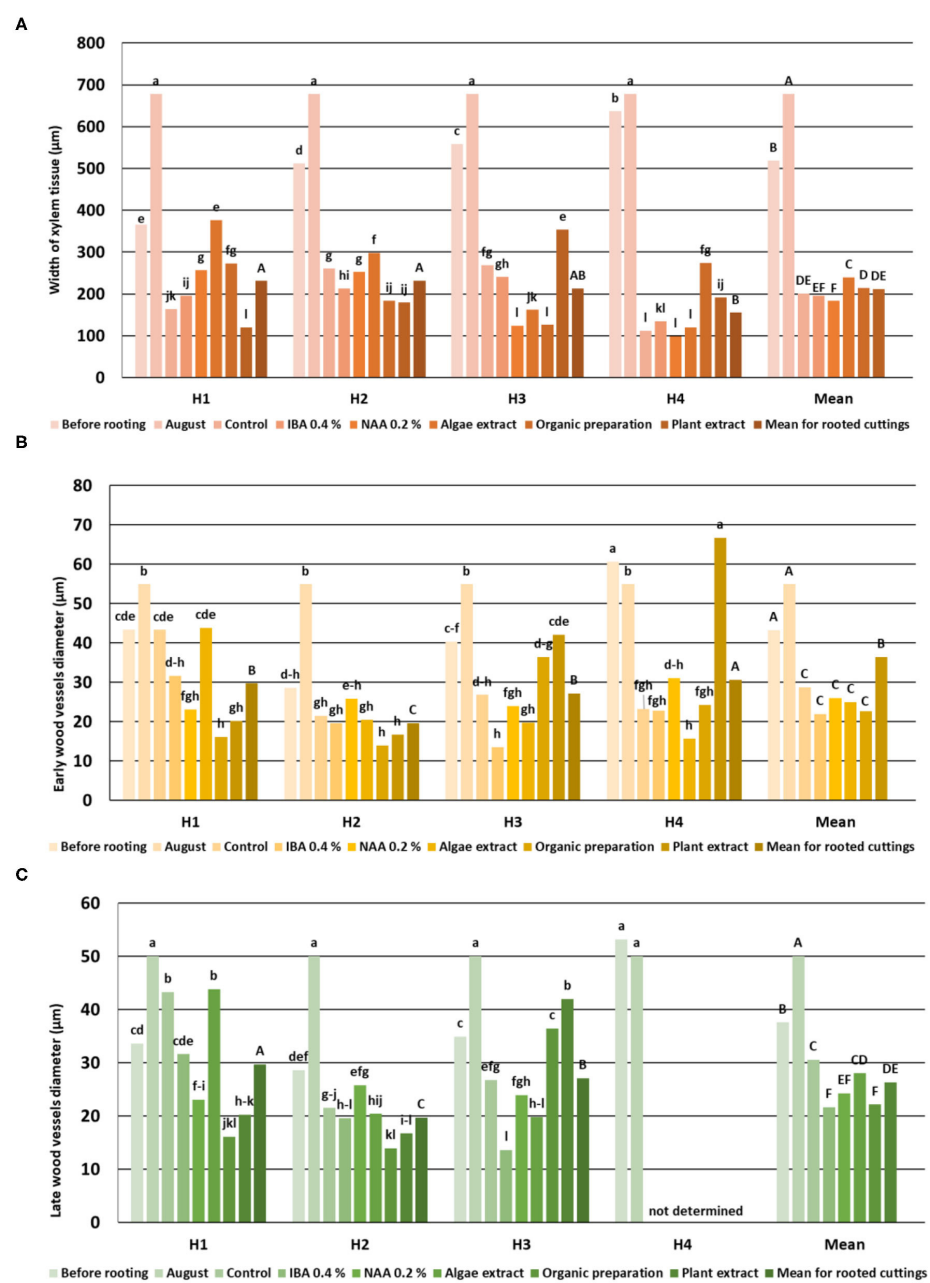
Width of *Rosa* “Hurdal” xylem tissue **(A)**, early- **(B)**, and late-wood **(C)** vessels in stems before rooting, cuttings after 24 days of the rhizogenesis process and in stems of stock plants cut in the first days of August. Control—the control cuttings; IBA 0.4%—Ukorzeniacz A_aqua_ (0.4% IBA); NAA, 0.2%—Ukorzeniacz B_aqua_ (0.2% NAA); Algae extract—watering with 0.6% Bio Rhizotonic (10 ml) after planting and 10 and 20 days later; Organic preparation—watering with 0.4% Root Juice™ (10 ml) after planting; Plant extract—watering with 0.02% Bio Roots (10 ml) after planting. H1−54 504 (flower buds still closed); H2−69 605 (full flowering; at least 50% of flowers open); H3−69 629 (end of flowering of the second-order stem—all petals have fallen); H4−70 701 [fruit set: beginning of hip (berry) growth on the main stem]. Different small letters indicate significant interactions between the phenological stage and treatment (two-way ANOVA). Different capital letters indicate significant differences between phenological stages. Duncan's test (α = 0.05) was used.

The vascular bundles were separated from the cortex sclerenchyma layer by irregularly arranged cells with very thickened walls and were different widths. Moreover, the ring of vascular bundles was partially irregular and with a few vascular bundles located outside. These irregular bundles were observed, especially in shoots H1 and H2. The structure visible on cross- and longitudinal sections of shoots in subsequent stages showed a progression of the lignification process (Figure 2). Stems collected from shoots in stages H3 and H4 had a wider wood region (Figure 4) where thick-walled fibers dominated, providing mechanical strength.

The width of xylem tissue increased with each fallow phenological stage. The vessels and tracheids of the xylem were located radially on the cross section in regular rows. No significant differences were found between the mean vessel diameter of early- and late-wood in H1, H2, and H3, while the means in H4 were significantly higher. There were a few cells of latewood in H1, mostly tracheids with thick walls. The layers of latewood were much wider in fallow phenological stages, and the vessels of latewood were significantly larger in diameter in H4 shoots. The width of xylem tissue increased in the shoots of stock plants until August, however without any increase to the diameter of the vessels. The last cells of visible latewood were mainly tracheids, with only a few large vessels (Figure 4).

The cambium layer in shoots before rooting consisted in three to four cells in H1, four to five in H3, and three to four in H4 (Figure 2). The cambium of stock plants in August was made up of five to six cells.

**Figure 5:**
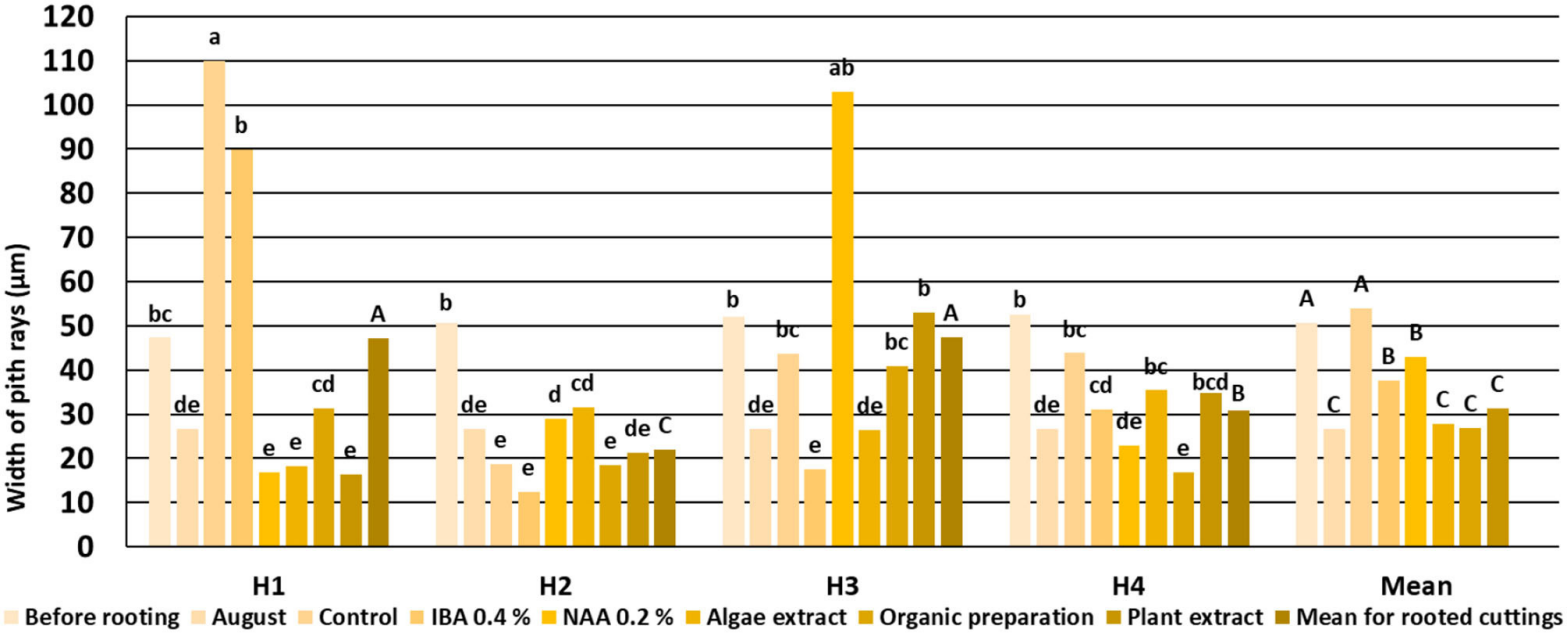
Width of *Rosa* “Hurdal” pith rays in stems before rooting; cuttings after 24 days of the rhizogenesis process; and stems of stock plants cut in first days of August. Control—the control cuttings; IBA, 0.4%—Ukorzeniacz A_aqua_ (0.4% IBA); NAA, 0.2%—Ukorzeniacz B_aqua_ (0.2% NAA); Algae extract—watering with 0.6% Bio Rhizotonic (10 ml) after planting and 10 and 20 days later; Organic preparation—watering with 0.4% Root Juice™ (10 ml) after planting; Plant extract—watering with 0.02% Bio Roots (10 ml) after planting. H1−54 504 (flower buds still closed); H2−69 605 (full flowering; at least 50% of flowers open); H3−69 629 (end of flowering of the second-order stem—all petals have fallen); H4−70 701 [fruit set: beginning of hip (berry) growth on the main stem]. Different small letters indicate significant interactions between the phenological stage and treatment (two-way ANOVA). Different capital letters indicate significant differences between phenological stages. Duncan's test (α = 0.05) was used.

The pith rays were narrow, made up of three to five rows of the cells. Numerous starch grains were visible in pith rays in comparison with their smaller number of companion cells of phloem, xylem fibers, and cortical parenchyma (Figure 2). The pith rays widths were similar throughout the four phenological stages (Figure 5).

The pith consisted of big living irregular cells, loosely stacked in starred form around small cells, with starch grains in cross sections and in rows on longitudinal sections (Figure 2).

### Rooting Enhancers Have a Varied Effect on Changes to the Anatomy of Stem Cuttings Harvested in Four Phenological Stages

**Figure 6:**
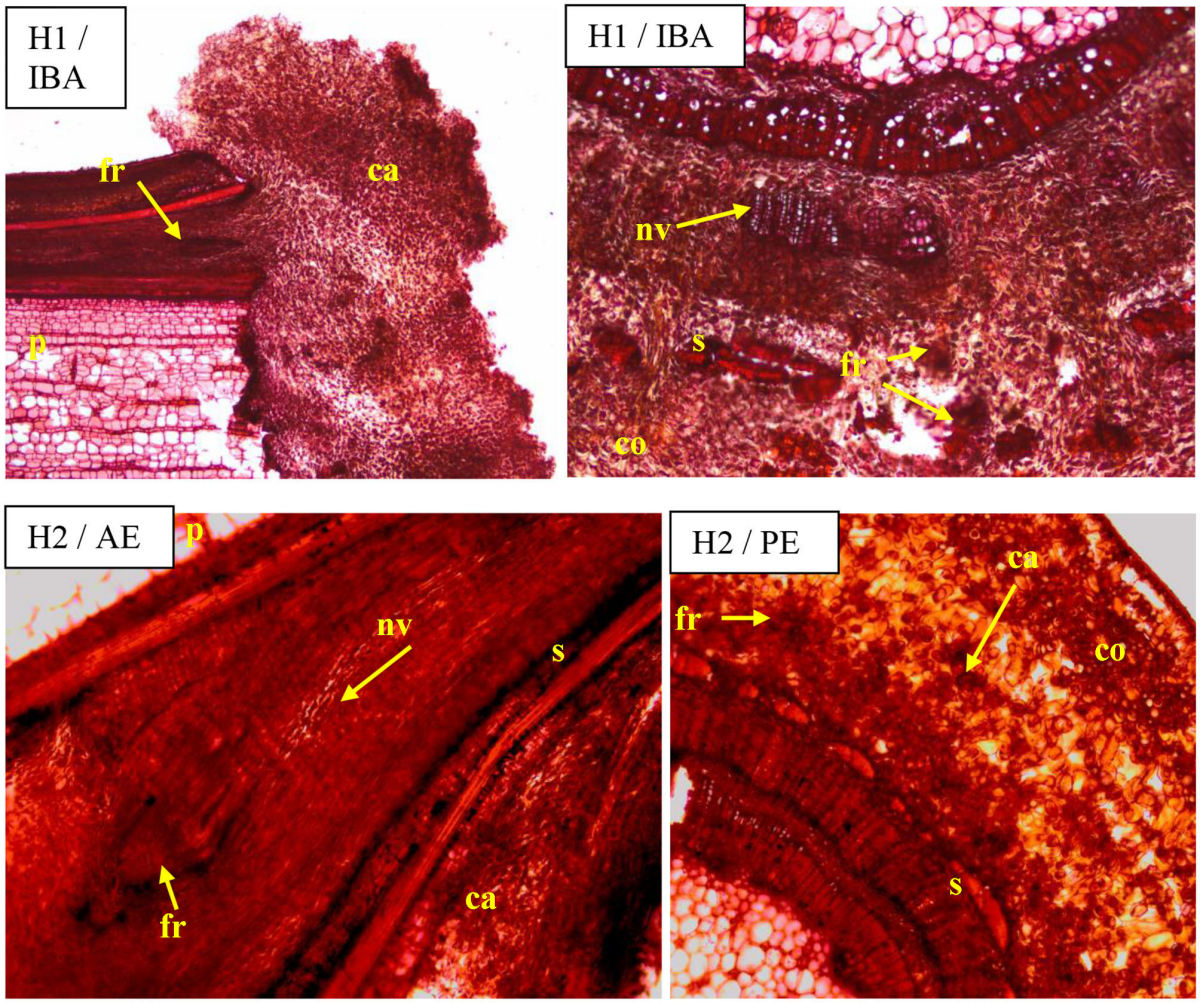
Transverse and longitudinal sections of the shoots after 25 days of rhizogenesis rooting in *Rosa* “Hurdal” in four phenological stages in BBCH scale (Meier et al., 2009): H1−54 504 (flower buds still closed); H2−69 605 (full flowering; at least 50% of flowers open). IBA—Ukorzeniacz A_aqua_ (0.4% IBA); AE—Algae extract—watering with 0.6% Bio Rhizotonic (10 ml) after planting, 10 and 20 days later; PE—Plant extract—watering with 0.02% Bio Roots (10 ml) after planting. Designation of signs: ca, callus; co, cortex; ph, phloem; p, pith; s, sclerenchyma; nv, new vascular bundles; fr, forming roots.

**Figure 7:**
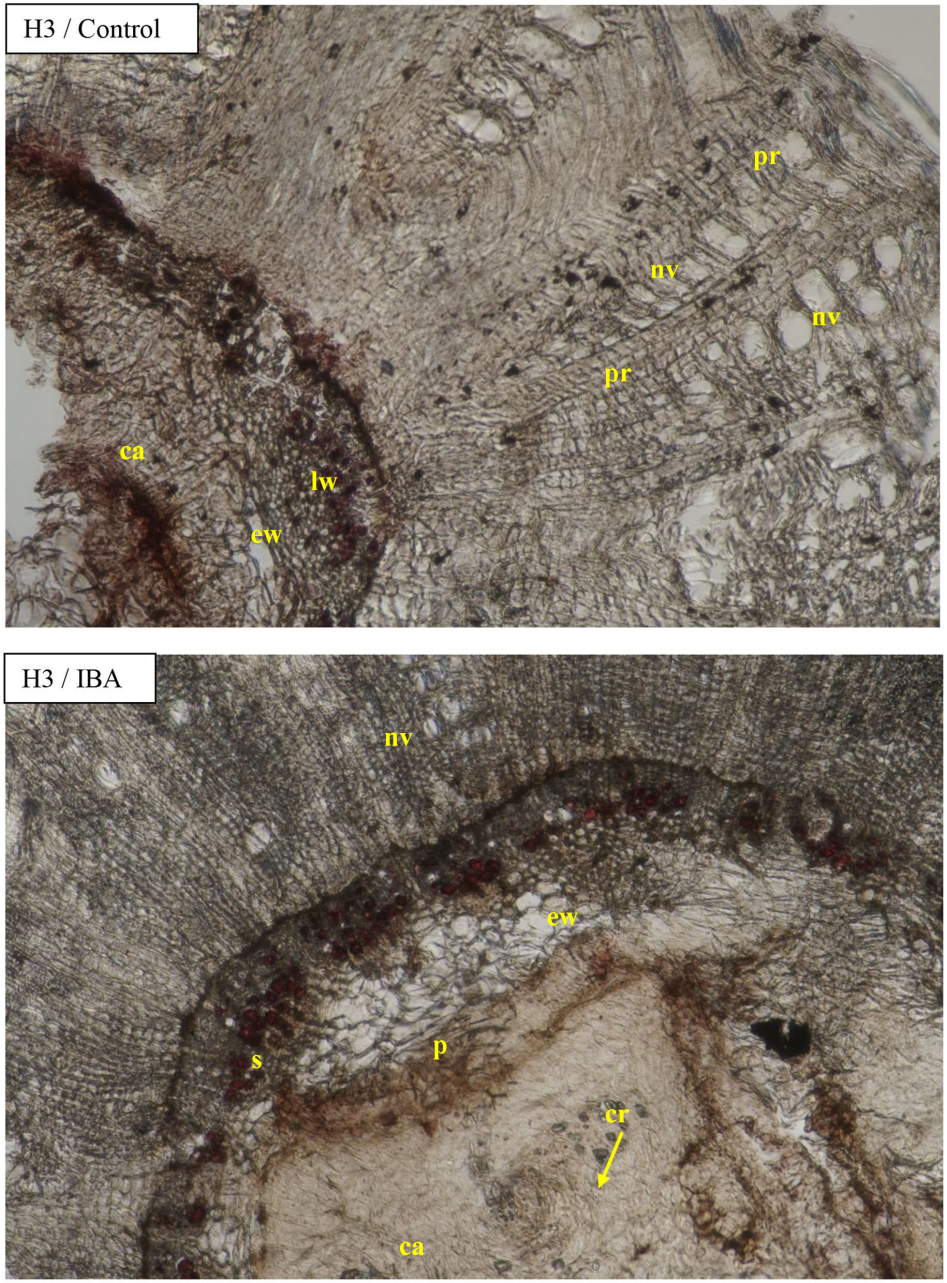
Transverse sections of the shoots after 25 days of the process of rhizogenesis rooting in *Rosa* “Hurdal” in phenological stages in BBCH scale (Meier et al., 2009) H3−69 629 (end of flowering of the second-order stem—all petals have fallen) in control—the control cuttings without rooting enhancers and after treatment IBA—Ukorzeniacz A_aqua_ (0.4% IBA). Designation of signs: ca, callus; c, cambium; co, cortex; cr, crystals; p, pith; pr, pith rays; s, sclerenchyma; nv, new vessels/vascular bundles; ew, earlywood; lw, latewood; fr, forming root.

**Figure 8:**
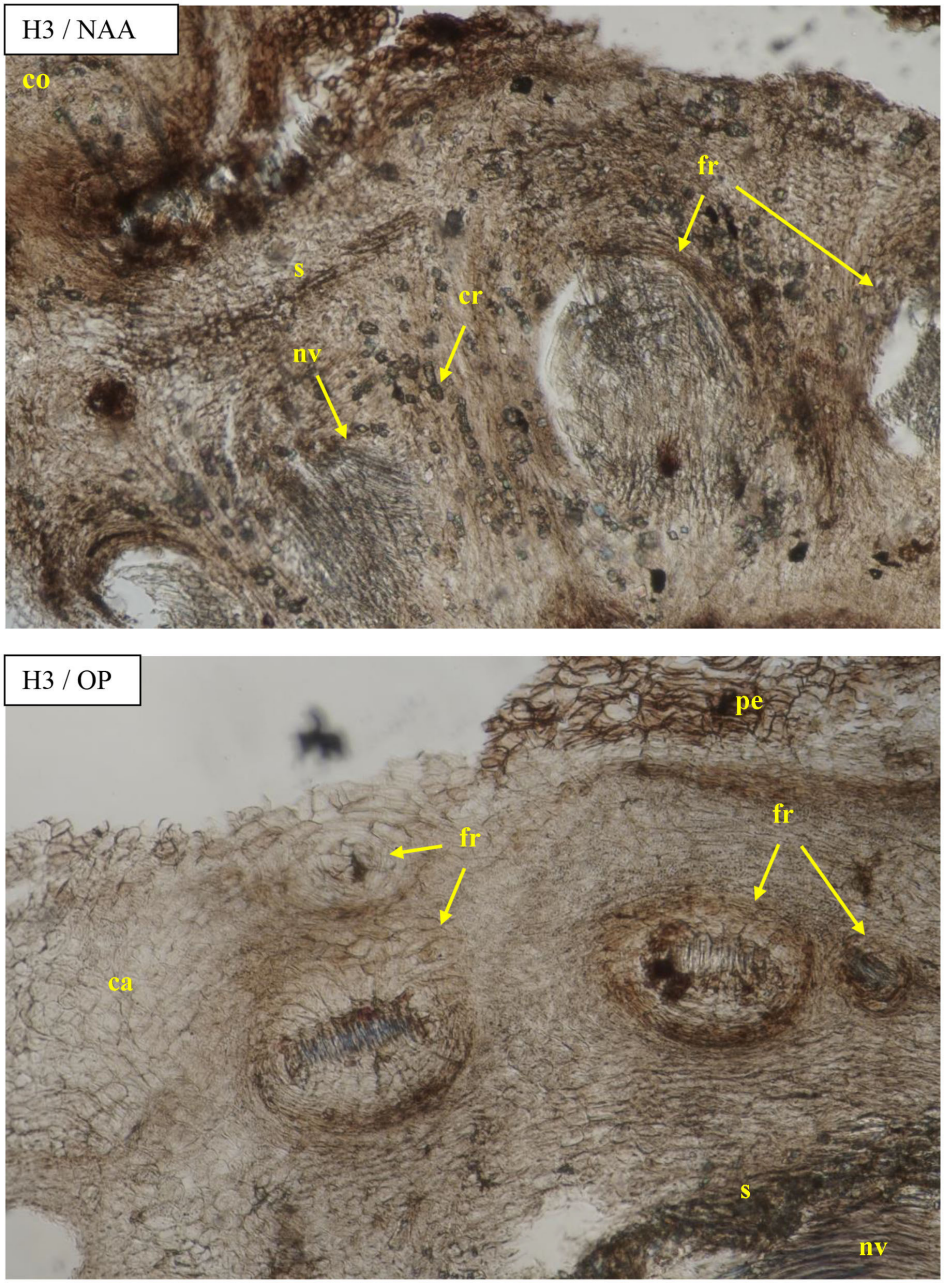
Transverse sections of the shoots after 25 days of the process of rhizogenesis rooting in *Rosa* “Hurdal” in the phenological stage in BBCH scale (Meier et al., 2009) H3−69 629 (end of flowering of the second-order stem—all petals fallen) in control—the cuttings after treatment of NAA—Ukorzeniacz B_aqua_ (0.2% NAA) and OP—Organic preparation—watering with 0.4% Root Juice™ (10 ml) after planting. Designation of signs: ca, callus; co, cortex; cr, crystals; pe, periderm; s, sclerenchyma; nv, new vessels/vascular bundles; fr, forming root.

Visible callus tissues appeared in the rhizogenesis process at the basal part of cuttings in the place of wounding after 10–14 days, and the first visible roots were spotted in week 4 near these places. On the cross sections, the roots most often appeared on the cambial zone near the pith rays and phloem. The forming roots were growing in outside, omitting the phloem and sclerenchyma layers. At the same time, numerous callus cells appeared, intensively filling the space between the first ring of xylem and sclerenchyma, and consequently growing outside through the cortex layer. The callus cells were also observed in the cambium zone and as a product of phellogen differentiation (Figure 6). The overgrowing of callus cells was also visible in the place of the pith on the base of the cutting (Figure 7). The roots probably also formed in the region of the phloem and pericycle, as was also observed after 12 weeks, without omitting the basal part of cuttings covered by a callus (Figure 8).

The width of the xylem tissue in control cuttings derived from shoots H1 was small compared to cuttings on which NAA preparation, Algae extract, or Organic preparation was used, and the width was even smaller in cuttings treated with Plant extract. This variable was also higher in control cuttings from shoots H2 for which Organic preparation or Plant extract was used as well as for cuttings obtained from shoots H3 treated with NAA, Organic preparation, and Plant extract. The width of xylem tissue in cuttings H4 was similar when using IBA and NAA preparations and Algae extract. The use of Organic preparation and Plant extract resulted in an increase in the width of xylem tissue. Xylem was dominated by large, thin-walled vessel elements of earlywood. However, with time, the wood cells became more regular in shape, smaller, thick-walled tracheids, with a tendency for the size of vessels to decrease in diameter in the cross section due to the use of rooting enhancers (Figure 4).

**Figure 9:**
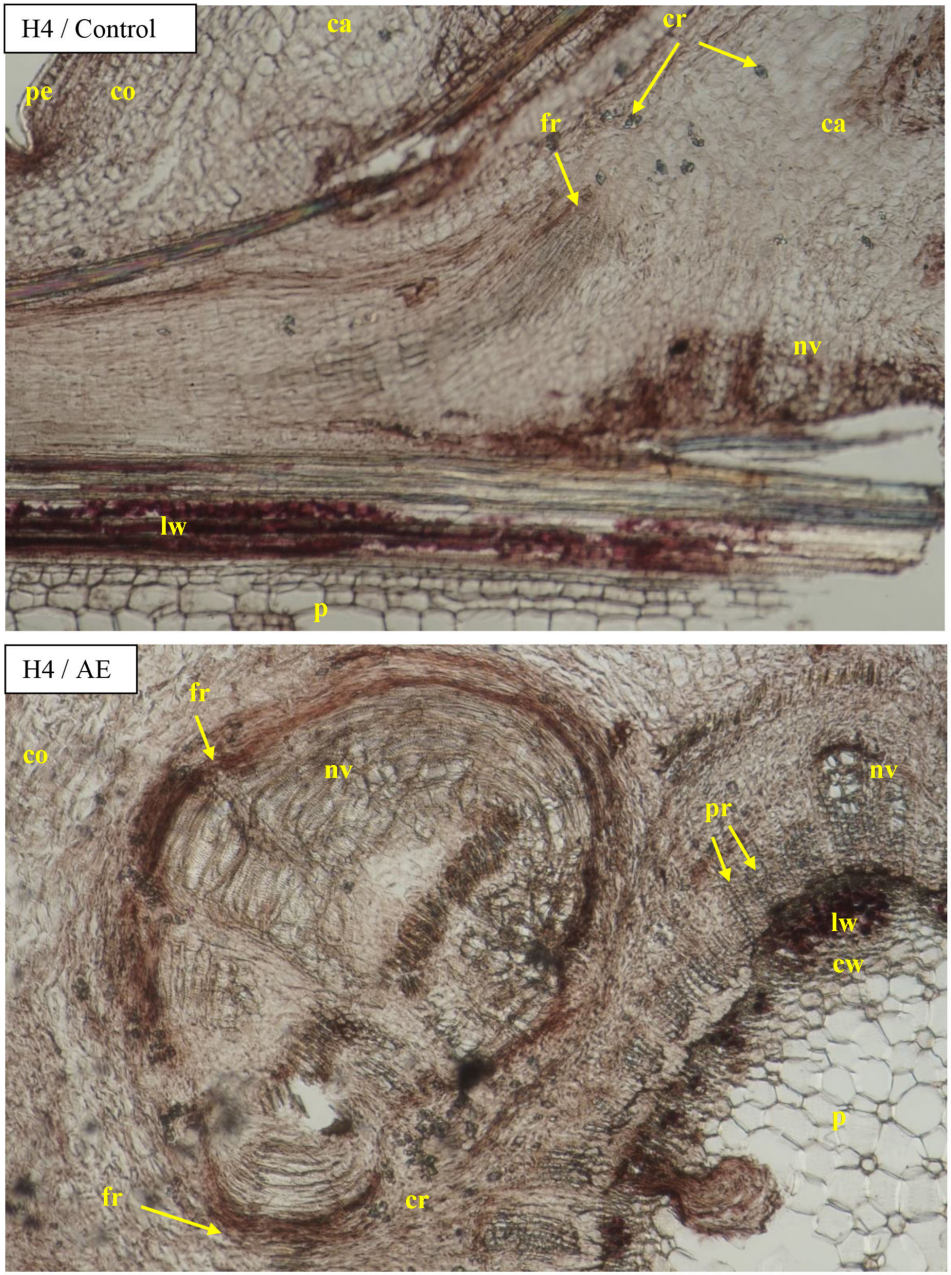
Transverse and longitudinal sections of the shoots after 25 days of the process of rhizogenesis rooting in *Rosa* “Hurdal” in the phenological stage in BBCH scale (Meier et al., 2009) H4−70 701 [fruit set: beginning of hip (berry) growth on the main stem] in control cutting and after treatment of AE—Algae extract—watering with 0.6% Bio Rhizotonic (10 ml) after planting, 10 and 20 days later. Designation of signs: pe, periderm; ca, callus; co, cortex; cr, crystals; p, pith; pr, pith rays; nv, new vessels/vascular bundles; ew, earlywood; lw, latewood; fr, forming root.

**Figure 10:**
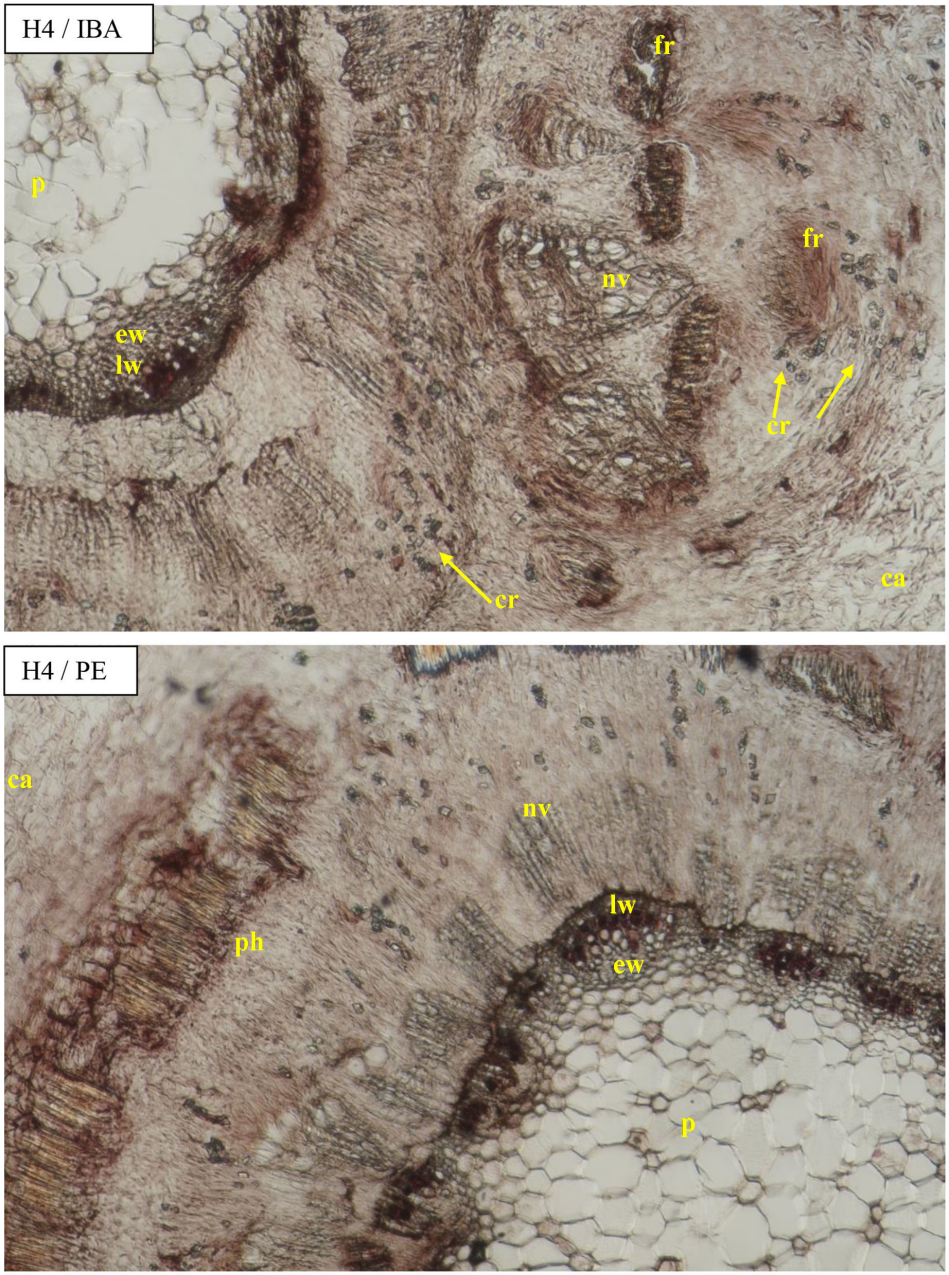
Transverse sections of the shoots after 25 days of the process of rhizogenesis rooting in *Rosa* “Hurdal” in the phenological stage in BBCH scale (Meier et al., 2009) H4−70 701 [fruit set: beginning of hip (berry) growth on the main stem] cutting after treatment of IBA—Ukorzeniacz A_aqua_ (0.4% IBA) and PE—Plant extract—watering with 0.02% Bio Roots (10 ml) after planting. Designation of signs: ca, callus; co, cortex; cr, crystals; p, pith; pr, pith rays; ph, phloem; nv, new vessels/vascular bundles; ew, earlywood; lw, latewood; fr, forming root.

The diameter of the vessels of early- and late-wood in H1 cuttings was higher in the control and after use of Algae extract as compared with other enhancers. In the case of H2 and H3 cuttings, the diameter of earlywood vessels was similar for all treatments. The diameter of latewood vessels of H2 after the use of Organic preparation was smaller than in the control cuttings. Compared to the control, H3 latewood vessels were larger in diameter after the use of an Organic preparation and Plant extract, and lower when using IBA and Algae extract. In the case of H4 cuttings, only the Plant extract affected the increase of earlywood diameter, while no latewood vessels were determined at this stage. The differentiation in new vessels and new ring had probably also inhibited (Figures 4, 9, 10).

The above results were compared with measurements of the xylem tissue in stock plants in August—the mean was higher than obtained by rhizogenezis in all stages H1–H4 (Figure 4). The cause for such results was the strong expansion of callus tissue compressing the xylem tissues of the first ring in rooted cuttings. This phenomenon probably also caused the separation of individual vascular bundles outside the first ring. A fragmentary new second ring with rows of vessels and tracheid cells was visible around and above the first ring (Figure 7).

The cambium zone in rooted cuttings was difficult to identify (Figures 6–10).

The process of rhizogenesis is characterized by intensive growth of the layer between the xylem and the cortex, especially high means were obtained for the cuttings H1, excluding those treated with Plant extract, for which the means were lowest. The growth of this layer decreased in control cuttings, following phenological stages from H2 up to H4, in which it was lowest. The preparations in H2–H4 cuttings had a different effect, with the tendency for NAA to decrease means (Figure 4).

The mean width of phloem tissue for rooted cuttings was highest in H1. The width of phloem tissue in H1 control-rooted cuttings was lower than after the use of rooting enhancers, excluding Plant extract. In the case of H4 rooted cuttings, the results were similar in all treatments, excluding Plant extract, for which the mean was higher (Figure 4).

The cortex grew intensively in H1 cuttings after the use of all rooting enhancers, and their widths were greater compared with the control. In the case of H2 cuttings, a higher mean as compared to the control was noticed as a result of the use of Plant extract only. In the case of H3 cuttings, the means after treatment by Organic preparation, Plant extract, and NAA were higher compared to the control. The use of IBA, NAA, and plant preparation affected the increase of the mean of the width of cortex tissue in H4 cuttings (Figure 4).

The widths of pith rays changed in the process of rhizogenesis, depending on the phenological stage and treatment. However, the means obtained for all cuttings indicated that the rooting enhancers caused a decrease in the width of pith rays compared to control cuttings (Figure 5).

The growth of cortex tissue and tissues between the cortex and the xylem was connected with the growth of callus tissue and auxiliary root formation visible on cross and longitudinal sections of the basal part of shoots (Figures 6–8).

### The Essential Role of the Phenological Stage of the Shoot and Plant Origin Preparations in Rooting the Cuttings of *Rosa* “Hurdal”

**Figure 11:**
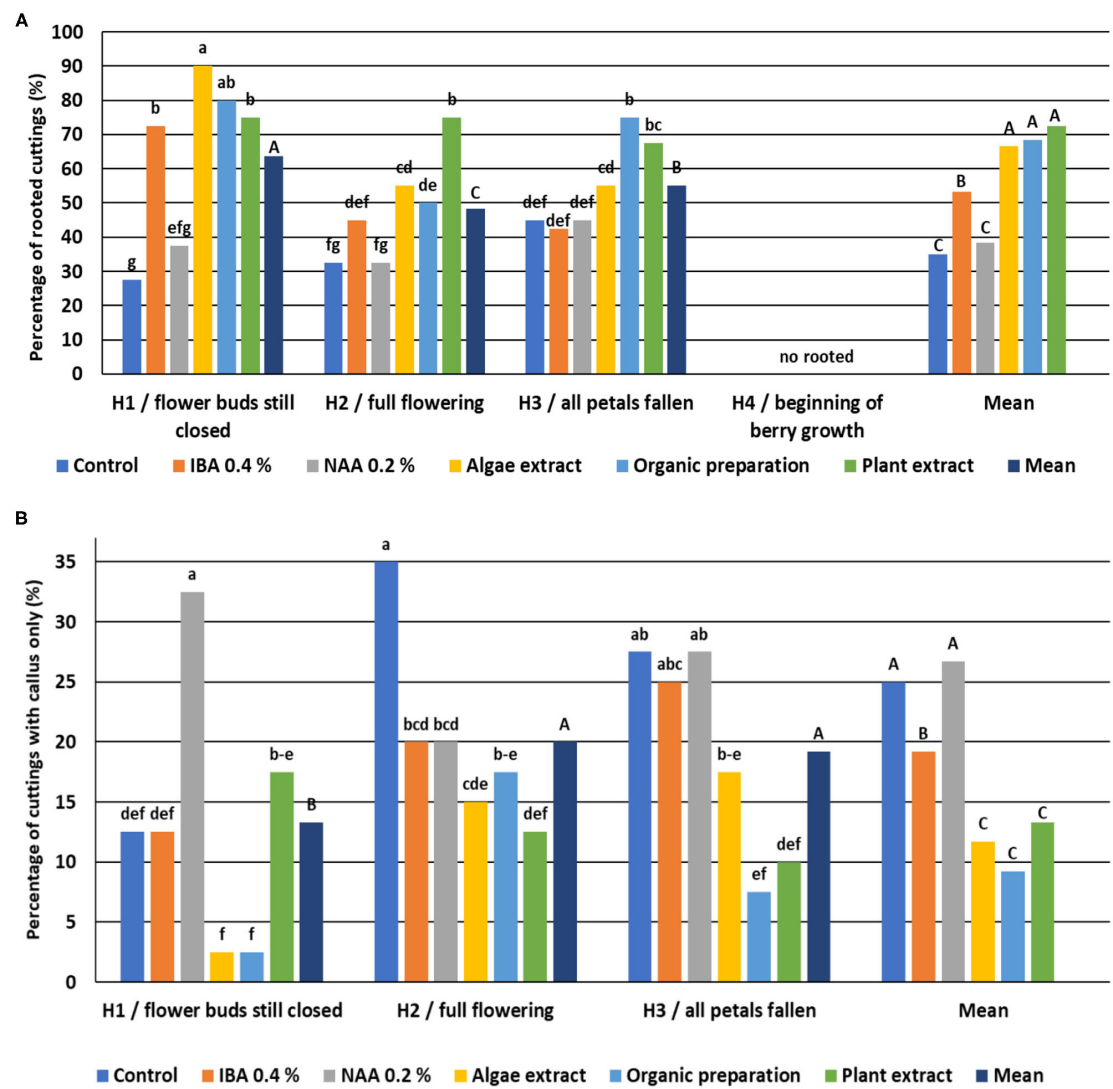
Percentage of rooted *Rosa* “Hurdal” cuttings **(A)** and cuttings with a callus only **(B)**. Control—the control cuttings; IBA, 0.4%—Ukorzeniacz A_aqua_ (0.4% IBA); NAA, 0.2%—Ukorzeniacz B_aqua_ (0.2% NAA); Algae extract—watering with 0.6% Bio Rhizotonic (10 ml) after planting and 10 and 20 days later; Organic preparation—watering with 0.4% Root Juice™ (10 ml) after planting; Plant extract—watering with 0.02% Bio Roots (10 ml) after planting. H1−54 504 (flower buds still closed); H2−69 605 (full flowering; at least 50% of flowers open); H3−69 629 (end of flowering of the second-order stem—all petals have fallen); H4−70 701 [fruit set: beginning of hip (berry) growth on the main stem]. Different small letters indicate significant interactions between the phenological stage and treatment (two-way ANOVA). Different capital letters indicate significant differences between phenological stages. Tukey's test (α = 0.05) was used.

The highest natural ability to root was shown for cuttings H3, and was lower for cuttings cut from shoots H1 and H2. The cuttings from shoots H4 did not root, and none of the rooting enhancers increased rooting ability (Figure 11).

In the case of cuttings H1, a 2- to 3-fold increase of rooting percentage was obtained through the use of IBA and preparations of plant origin compared to the control cuttings and treated NAA. The lower (control, IBA, and plant origin preparations) or similar (NAA) percentage of cuttings with a callus only was noticed. The use of rooting enhancers for cutting H2 decreased the percentage of cuttings with the callus only. The rooting percentage increased after the use of plant origin preparations compared to the control cuttings. Organic preparation and Plant extract had a similar effect on H3 cuttings (Figure 11).

**Figure 12:**
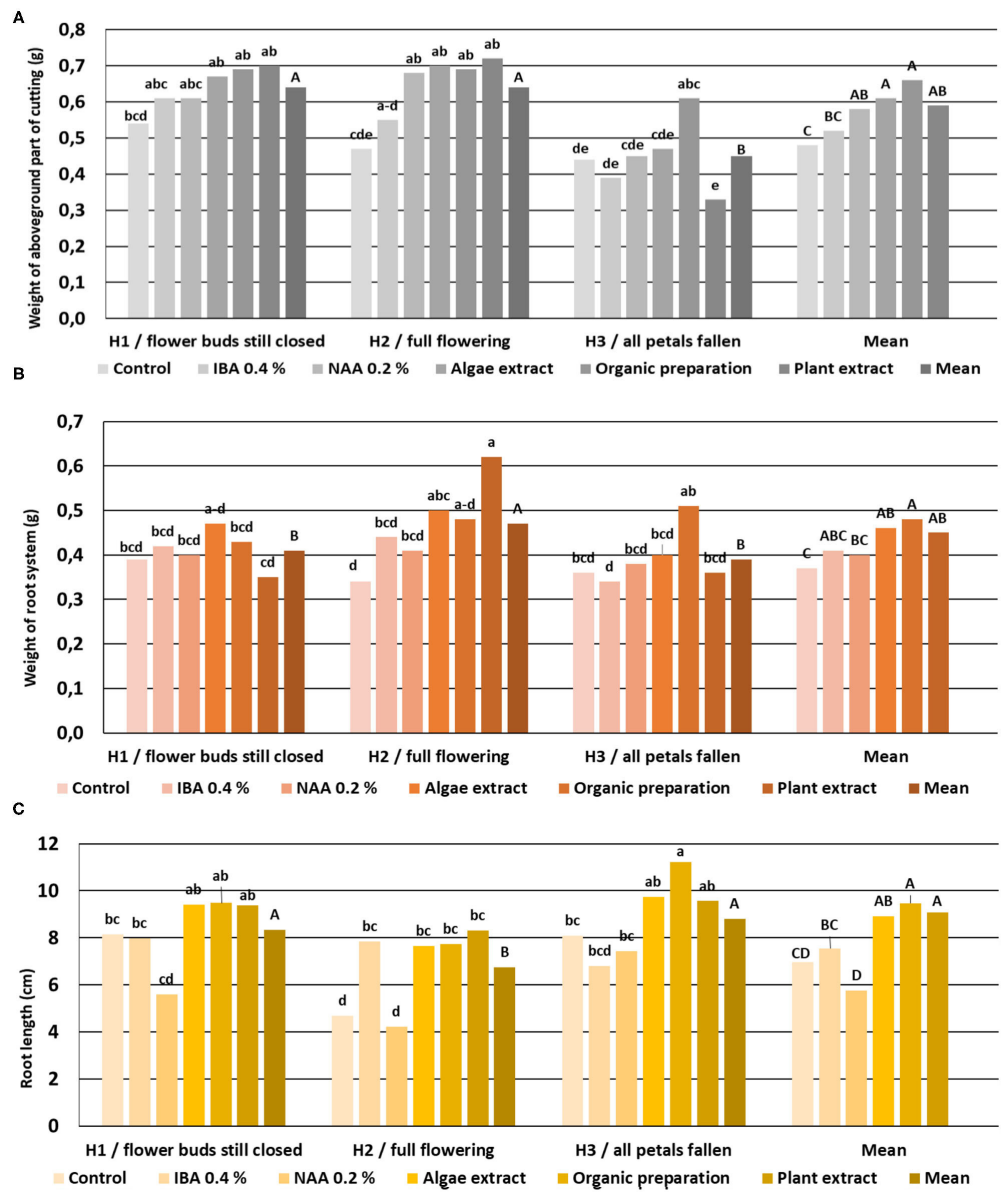
The root length **(C)**, weight of the aboveground part **(A)**, and root system **(B)** in rooted cuttings of *Rosa* “Hurdal.” Control—the control cuttings; IBA, 0.4%—Ukorzeniacz A_aqua_ (0.4% IBA); NAA, 0.2%—Ukorzeniacz B_aqua_ (0.2% NAA); Algae extract—watering with 0.6% Bio Rhizotonic (10 ml) after planting, 10 and 20 days later; Organic preparation—watering with 0.4% Root Juice™ (10 ml) after planting; Plant extract—watering with 0.02% Bio Roots (10 ml) after planting. H1−54 504 (flower buds still closed); H2−69 605 (full flowering; at least 50% of flowers open); H3−69 629 (end of flowering of the second-order stem—all petals have fallen); H4−70 701 [fruit set: beginning of hip (berry) growth on the main stem]. Different small letters indicate significant interactions between the phenological stage and treatment (two-way ANOVA). Different capital letters indicate significant differences between phenological stages. Tukey's test (α =0.05) was used.

The rooting enhancers did not affect the root length, the weight of the root system, or the aboveground part of cuttings H1. In the case of H2 stage cuttings, the use of NAA preparation and all the preparation of plant origin increased the weight of the aboveground part of the plants, while the Algae extract and Plant extract affected the weight of the root systems. The use of IBA and all preparation of plant origin increased the root length. The use of an Organic preparation resulted in higher root length, weight of the aboveground part as compared to the remaining combination of rooted H3-stage cuttings. In the case of cuttings derived from shoots at this phenological stage, none of the rooting enhancers affected the weight of the root system (Figure 12).

### Changes in Anatomy Structure Contra Rooting Percentage Results in the Propagation of *Rosa* “Hurdal”

Correlation analysis for the rooted cuttings in all phenological stages (H1–H3) showed that an increase in rooting percentage was only strictly connected to a decrease of the percentage of cuttings with a callus only. Moreover, a higher percentage of rooted cuttings correlated positively with a higher root length, weight of the root system, and aboveground part of cuttings, and also higher mean width of tissues between the xylem and cortex, a greater diameter of early- and late-wood vessels, and wider phloem and cortex layers. In the case of the percentage of cuttings with a callus only, higher results were obtained when the root length, weight of the root system of cuttings, width of tissues between xylem and cortex, and the diameter of early- and late-wood vessels were decreased and the phloem layer was getting narrower. The increase in length root correlated with the increase in diameter of early- and late-wood vessels, and the wider phloem layer. Correlation analysis showed a positive relationship between the weight of the root system and the aboveground part of cuttings. The anatomy parameters were also interconnected (Table 2).

**Table 2:** Effect correlations between measured parameters of growth and anatomical structure in *Rosa* “Hurdal” after 25 days of rooting cuttings in all phases taken together.

**Variable**	**Rooting percentage**	**Percentage of cuttings with callus only**	**Root length**	**Fresh weight of root system**	**Fresh weight of above ground part**	**Width of xylem tissue**	**Width of tissues between xylem and cortex**	**Early wood vessel diameter**	**Late wood vessel diameter**	**Width of pith rays**	**Width of phloem tissue**	**Width of cortex tissue**
	**1**	**2**	**3**	**4**	**5**	**6**	**7**	**8**	**9**	**10**	**11**	**12**
1	1.00											
2	−0.63^***^	1.00										
3	0.44^**^	−0.49^**^	1.00									
4	0.10^*^	−0.07	0.03	1.00								
5	0.15^*^	−0.11^*^	−0.01	0.48^**^	1.00							
6	0.07	−0.06	−0.06	0.02	−0.03	1.00						
7	0.17^*^	−0.21^*^	0.05	0.02	0.08^*^	0.30^**^	1.00					
8	0.10^*^	−0.17^*^	0.12^*^	−0.03	−0.06	0.14^*^	0.34^**^	1.00				
9	0.10^*^	−0.17^*^	0.12^*^	−0.03	−0.06	0.14^**^	0.34^**^	0.99^*****^	1.00			
10	−0.22^*^	−0.07	0.03	−0.05	−0.05	−0.15^**^	0.24^*^	0.33^**^	0.33^**^	1.00		
11	0.25^*^	−0.25^*^	0.14^*^	0.02	0.09^*^	0.26^**^	0.76^****^	0.35^**^	0.35^**^	0.12^*^	1.00	
12	0.23^*^	−0.05	0.07	−0.02	0.09^*^	0.03	0.25^*^	0.07	0.07	−0.15^*^	0.14^*^	1.00

*Marked correlations are significant at p < 0.05. Correlation significance*:

* *0.08–0.29—very low*;

** *0.30–0.49—low*;

*** *0.50–0.69—restrained*;

**** *0.70–0.89—high*;

***** *>0.90—very high*.

Correlation analysis for cuttings derived from shoots with flower bud closed H1 showed a relationship between rooting percentage and estimated parameters in subsequent treatments. A larger diameter of latewood vessels in shoots before rooting was correlated with rising rooting percentage in control cuttings, treated with Algae extract, and Organic preparation, and decreasing rooting percentage with the use of NAA and Plant extract. A higher rooting percentage correlated with increase in early- and late-wood vessel diameters in cuttings 25 days after rooting, treated with Algae extract, and Organic preparation, while decreasing these parameters and the width of phloem tissue in control cuttings. The increase in rooting percentage was correlated with an increase in the percentage of cuttings with a callus only in the control, while with a decrease in this parameter when IBA, NAA, Algae extract, and Plant extract was used. An increase in rooting percentage was connected with an increase in fresh weight of the aboveground part and root system of cuttings after the use of Algae extract, while a decrease was associated with the use of Organic preparation. The higher rooting percentage was correlated with an increase in weight of the root system after the use of IBA and of the aboveground part in control cuttings. Moreover, the increase of rooting percentage correlated with the increase of root length in control and, after the use of all rooting enhancers, excluding Algae extract (Table 3).

**Table 3:** Effect correlations between percentage of rooted cuttings and measured parameters of growth and anatomical structure in *Rosa* “Hurdal” before and after 25 days of rooting cuttings derived from shoots H1, with flower buds closed (BBCH 54 504).

	**Percentage of rooted cuttings**					
**Variable**	**Control**	**IBA**	**NAA**	**Algae extract**	**Organic preparation**	**Plant extract**
		**0.4%**	**0.2%**	**3 × 0.6%**	**0.4%**	**0.02%**
SD	33.31	18.08	8.40	12.40	7.16	23.20
Percentage of cuttings with callus only	0.29^*^	−0.88^****^	−1.00^*****^	−0.94^*****^	0.00	−1.00^*****^
Root length	0.24^*^	0.44^**^	0.88^****^	−0.28^*^	0.22^*^	0.65^***^
Fresh weight of root system	0.02	0.33^**^	0.07	0.15^*^	−0.49^**^	−0.01
Fresh weight of aboveground part	0.33^**^	−0.02	0.01	0.17^*^	−0.42^**^	0.01
Width of xylem tissue	−0.04	−0.01	0.01	0.01	−0.05	−0.10
Width of tissues between xylem and cortex	0.00	0.00	0.00	0.00	0.00	0.00
Earlywood vessels diameter	−0.12^*^	0.00	0.00	0.25^*^	0.17^*^	0.00
Latewood vessels diameter	−0.12^*^	0.00	0.00	0.25^*^	0.17^*^	0.00
Width of pith rays	0.00	0.00	0.00	0.00	0.00	0.00
Width of phloem tissue	−0.11^*^	0.02	0.03	−0.03	0.04	0.00
Width of cortex	0.00	0.00	0.00	0.00	0.00	0.00
**Before rooting**						
Width of xylem tissue	0.00	0.00	0.00	0.00	0.00	0.00
Earlywood vessel diameter	−0.03	0.01	0.02	−0.03	−0.06	0.02
Latewood vessel diameter	0.14^*^	−0.05	−0.11^*^	0.15^*^	0.25^*^	−0.08^*^
Width of pith rays	0.00	0.00	0.00	0.00	0.00	0.00
Width of phloem tissue	0.04	−0.01	−0.03	0.04	0.07	−0.02
Width of cortex	−0.04	0.01	0.03	−0.04	−0.07	0.02
Width of early wood tissue	0.00	0.00	0.00	0.00	0.00	0.00
Width of late wood tissue	0.00	0.00	0.00	0.00	0.00	0.00

*Marked correlations are significant at p < 0.05. Correlation significance*:

* *0.08–0.29—very low*;

** *0.30–0.49—low*;

*** *0.50–0.69—restrained*;

**** *0.70−0.89—high*;

***** *>0.90—very high*.

The rooting percentage of H2 stage cuttings was correlated with the anatomical structure parameters of the shoots before rooting. A higher rooting percentage of control cuttings correlated with an increase in the width of xylem tissue and in the diameter of early- and late-wood vessels, but with a decrease in the width of phloem tissue in shoots before rooting cuttings. The increase in rooting percentage by the use of NAA and plant origin preparations correlated with a decrease in the width of xylem tissue in shoots before rooting. The increase in rooting percentage by the use of IBA was correlated with the increase in the diameter of early- and late-wood vessels, but a decrease in the width of phloem and cortex tissue before rooting. In the case of the use of Organic preparation and Plant extract, the increase of the width of phloem and cortex tissue and the decrease of the diameter of early- and late-wood vessels before rooting positively correlated with rooting percentage. After 25 days of rooting, an increase in the early- and late-wood vessel diameter and a decrease in the width of phloem tissue through the use of Organic preparations were correlated with the increase of rooting percentage. Correlation analysis for H2 cuttings showed a relationship between the increase of rooting percentage and the decrease of the percentage of cuttings with a callus only in control cuttings and after the use of NAA preparation, but with the increase by the use of Organic preparation and Plant extract. An increase in rooting percentage was correlated with an increase in the fresh weight of the root system and aboveground part of the rooted cuttings in the case of control cuttings and after the use of IBA. A decrease of the weight of the root system by the use of NAA caused the rooting percentage to increase. The increase in rooting percentage was correlated with an increase in the root length in the case of control cuttings and after the use of IBA, NAA, Algae, and Plant extract (Table 4).

**Table 4:** Effect correlations between percentage of rooted cuttings and measured parameters of growth and anatomical structure in *Rosa* “Hurdal” before and after 25 days of rooting cuttings derived from shoots H2, in full flowering; at least 50% of flowers open (BBCH 69 605).

	**Percentage of rooted cuttings**					
**Variable**	**Control**	**IBA**	**NAA**	**Algae extract**	**Organic preparation**	**Plant extract**
		**0.4%**	**0.2%**	**3 × 0.6%**	**0.4%**	**0.02%**
SD	13.56	5.06	23.07	5.06	7.16	11.32
Percentage of cuttings with callus only	−0.75^****^	0.00	−0.62^***^	0.00	0.43^**^	−0.94^*****^
Root length	0.20^*^	0.50^**^	0.50^**^	0.70^****^	0.04	0.28^*^
Fresh weight of root system	0.08^*^	0.34^**^	−0.13^*^	−0.04	−0.05	−0.14
Fresh weight of aboveground part	0.09^*^	0.16^*^	0.08^*^	−0.07	0.05	0.04
Width of xylem tissue	−0.11^*^	0.03	0.04	0.02	0.06	−0.08
Width of tissues between xylem and cortex	0.00	0.00	0.00	0.00	0.00	0.00
Earlywood vessels diameter	−0.07	−0.10	0.04	0.00	0.18^*^	−0.09^*^
Latewood vessels diameter	−0.07	−0.10	0.04	0.00	0.18^*^	−0.09^*^
Width of pith rays	0.00	0.00	0.00	0.00	0.04	0.00
Width of phloem tissue	−0.02	−0.26	0.00	0.00	−0.20^*^	0.02
Width of cortex	0.00	0.00	0.00	0.00	0.00	0.00
**Before rooting**						
Width of xylem tissue	0.18^*^	0.02	−0.18^*^	−0.19^*^	−0.15^*^	−0.11^*^
Earlywood vessel diameter	0.09^*^	0.45^**^	0.05	0.00	−0.32^**^	−0.40^**^
Latewood vessel diameter	0.09^*^	0.45^**^	0.05	0.00	−0.32^**^	−0.40^**^
Width of pith rays	0.00	0.00	0.00	0.00	0.00	0.00
Width of phloem tissue	−0.08^*^	−0.42^**^	−0.05	0.00	0.29^*^	0.37^**^
Width of cortex	−0.05	−0.24^*^	−0.03	0.00	0.17^*^	0.22^*^
Width of early wood tissue	0.00	0.00	0.00	0.00	0.00	0.00
Width of late wood tissue	0.00	0.00	0.00	0.00	0.00	0.00

*Marked correlations are significant at p < 0.05. Correlation significance*:

* *0.08–0.29—very low*;

** *0.30–0.49—low*;

*** *0.50–0.69—restrained*;

**** *0.70−0.89—high*;

***** *>0.90—very high*.

In the case of cuttings derived from shoots immediately after petals had fallen (H3), correlations between rooting percentage and parameters of growth, as well as anatomical structure, were proved. Increasing rooting percentage was shown to correlate with the width of xylem tissue in shoots before rooting, where increase in the case of control cuttings and decrease in rooting enhancers were used. The larger diameter of earlywood in shoots before rooting correlated with rising rooting percentage for control cuttings and cuttings treated with Plant extract and decreasing in the case of treatment with NAA and Organic preparations. A decrease in the width of cortex tissue in control cuttings and in the latewood vessel diameter in shoots before rooting correlated with an increase in rooting percentage for cuttings treated with IBA, NAA, and Algae extract. The increasing of width of the cortex layer in shoots before rooting was positively connected with rooting percentage where NAA, Algae extract, and Organic preparations were used. The increasing of width of the xylem tissue, early- and late-wood diameters, and phloem tissue after 25 days of rooting was correlated with higher rooting percentage in the case of control cuttings. Moreover, the rise in the diameter of early- and latewood vessels after 25 days in cuttings treated with Algae extract and Organic preparation correlated positively with rooting percentage. In the case of cuttings rooted with the use of a Plant extract, a correlation was found between the increase of rooting percentage and the decrease of the early- and late-wood vessel diameter and width of pith rays and cortex tissue in cuttings after 25 days of rooting. The increase of rooting percentage was strictly correlated with a decrease in percentage of cuttings with a callus only both in control cuttings and after the use of any rooting enhancer. Moreover, the increase of rooting percentage correlated with the decrease of fresh weight of the root system for the control cuttings and treated auxins, while with the increase of the weight of the aboveground parts of cuttings treated with the NAA. Additionally, the rooting percentage increased with increase in the root length in the case of control cuttings and after the use of IBA, NAA, Algae, and Plant extract (Table 5).

**Table 5:** Effect correlations between percentage of rooted cuttings and measured parameters of growth and anatomical structure in *Rosa* “Hurdal” before and after 25 days of rooting cuttings derived from shoots H3, immediately after petals have fallen (BBCH 69 629).

	**Percentage of rooted cuttings**					
**Variable**	**Control**	**IBA**	**NAA**	**Algae extract**	**Organic preparation**	**Plant extract**
		**0.4%**	**0.2%**	**3 × 0.6%**	**0.4%**	**0.02%**
SD	16.74	24.15	16.79	15.19	15.19	13.16
Percentage of cuttings with callus only	−0.64^***^	−0.99^*****^	−0.98^*****^	−0.51^***^	−0.70^****^	−0.19^*^
Root length	0.29^*^	0.53^***^	0.40^**^	0.12^*^	0.07	0.10^*^
Fresh weight of root system	−0.12^*^	−0.17^*^	−0.16^*^	−0.01	0.05	−0.04
Fresh weight of aboveground part	−0.04	−0.01	0.22^*^	0.06	0.06	0.03
Width of xylem tissue	0.15^*^	0.07	−0.15^*^	−0.06	0.01	0.00
Width of tissues between xylem and cortex	0.00	0.00	0.00	0.00	0.00	0.00
Earlywood vessels diameter	0.13^*^	0.00	−0.04	0.08^*^	0.09^*^	−0.26^*^
Latewood vessels diameter	0.13^*^	0.00	−0.04	0.08^*^	0.09^*^	−0.26^*^
Width of pith rays	0.00	0.00	0.00	0.00	0.00	−0.96^*****^
Width of phloem tissue	0.18^*^	0.00	0.04	0.00	0.00	0.00
Width of cortex	−0.10	0.00	0.00	0.00	0.00	−0.15^*^
**Before rooting**						
Width of xylem tissue	0.33^**^	−0.09^*^	−0.26^*^	−0.24^*^	−0.17^*^	−0.10^*^
Earlywood vessel diameter	0.15^*^	−0.05	−0.15^*^	0.00	−0.33^**^	0.48^**^
Latewood vessel diameter	0.05	−0.13^*^	−0.13^*^	−0.13^*^	−0.07	0.07
Width of pith rays	0.00	0.00	0.00	0.00	0.00	0.00
Width of phloem tissue	0.00	0.00	0.00	0.00	0.00	0.00
Width of cortex	−0.71^****^	0.05	0.46^**^	0.32^**^	0.47^**^	−0.01
Width of early wood tissue	0.00	0.00	0.00	0.00	0.00	0.00
Width of late wood tissue	0.00	0.00	0.00	0.00	0.00	0.00

*Marked correlations are significant at p < 0.05. Correlation significance*:

* *0.08–0.29—very low*;

** *0.30–0.49—low*;

*** *0.50–0.69—restrained*;

**** *0.70−0.89—high*;

***** *>0.90—very high*.

## Discussion

Changes in the seasonal cambial activity and morphophysiological status of stock plants affected the adventitious root formation of cuttings of *Artemisia arborescens* (Fascella et al., 2012), *Ficus infectoria* (Anand and Heberlein, 1975), *Juniperus* L. (Torchik, 2015), olive (Abousalim et al., 1993), and roses (Monder et al., 2017). The different periods of delivery and rooting of cuttings suggested changes in various groups of roses (Hoşafçi et al., 2005; Pihlajaniemi et al., 2005; Monder et al., 2016).

The 1-year shoots of *R*. “Hurdal” used for cuttings in all phenological stages before and after rhizogenesis were characterized by an anatomical structure typical for woody plants of *Rosaceae* and the genus *Rosa* (Zhang, 1992). The diameters of the vessels measured by Zhang (1992) were 70–400 (mostly 114–192) μm, 40–96% solitary, round to oval, or angular, whereby the tangential diameter of earlywood vessels was 35–155 (40–250) μm and tangential latewood vessels−19–52 (15–130) μm. The means of this parameter were low for *R*. “Hurdal.” The mean diameter of the earlywood vessel before rooting depends on the phenological stage and reaches a mean from 28.66 μm in H2 to 60.59 μm in H4. The latewood vessel diameter showed a tendency to increase and reach a mean from 28.86 μm in H2 to 53.2 μm in H4. Low values of the diameter can limit the transport of water (Rosell et al., 2017), and, additionally, single-node leafy stem cuttings have limited storage capacity due to the small size of the shoot, which hinders the survival of the cuttings (Costa et al., 2007). However, the vessel diameter in woody plants is affected by many factors (Rosell et al., 2017). Moreover, the tracheids of *R*. “Hurdals” had thick walls and a very small diameter. However, the callus in *Rosa* Madelon® had differentiated vessels that can contribute to water transport before new roots take over this function (Costa et al., 2007).

The natural reaction of a cambium to wounding caused by cutting is a callus formation at the base, both in woody (Strzelecka, 2017) and herbaceous plants (Wróblewska, 2013). The callus proliferation precedes root initiation. The proliferation of parenchyma or phloem tissue (callus) is crucial to root formation and better rooting of shoot cuttings. The new parenchyma tissue (callus) can grow rapidly through cambial meristematic activity before forming adventitious roots, as in the stem cuttings of *Rosa* Madelon® (Costa et al., 2007). The adventitious roots formed by *Rosa rugosa* stem cuttings are preceded by callus tissue at the cut surface of the stem base (Fouda and Schmidt, 1994). A callus can prevent the entry of pathogenic organisms at the wound (Cline and Neely, 1983), affect positively the hydration of a cutting (Scagel, 2004), and reduce susceptibility to stem rot (Howard and Harrison-Murray, 1995). However, callus overgrowth could be an unfavorable phenomenon in *R*. “Hurdal,” and result in a high percentage of cuttings with calluses only. Vessel measurement after 25 days suggested a decrease in such cuttings, probably because their tissues had been squeezed by growing callus cells; however, the vessel diameter of latewood in H4 was not determined due to the lack of unambiguous differentiation between the cells. The effects of preparations were different and depended on the phenological stage in *R*. “Hurdal” as in *Rosa beggeriana* “Polstjårnan” and *Rosa helenae* “Semiplena” (Monder et al., 2019). The IBA and NAA preparations decreased the width of xylem tissue, probably because of overgrowth of callus cells.

The shoots of *R*. “Hurdals” grew intensively up to the appearance of flower buds and harvest time for cuttings. The lignifying process of the shoots progressed and stem lignification and regular tissue arrangement were characteristics in rooted cuttings during all of the H1–H4 phases. The same observations were also visible on both once-flowering *R*. “Polstjårnan” and *R*. “Semiplena” (Monder et al., 2019).

The experiment conducted on *R*. “Hurdals” show the crucial role of the phenological stage of shoots of the stock plant in which the cuttings were prepared, both in terms of propagation and anatomical changes. The changes to anatomical structure in subsequent phenological stages of flowering before the cuttings were prepared affected the response to rooting enhancers and consequently the effectiveness of rhizogenesis.

The experiment also shows that the changes in anatomical structure before rooting cuttings affected the changes in anatomy structure in response to IBA, NAA, and plant origin preparations in the process of rhizogenesis. It suggested that the specific anatomic structure of shoots before rooting led to favorable or unfavorable changes caused by rooting enhancers, including plant origin preparations. Changes in the structure of the cambium zone, where the cells differentiate, are of particular importance. Changes to cortex tissues, where the cells of phellogen can also differentiate, are equally significant.

The overgrowth of a callus may squeeze the first ring of xylem tissue, consequently decreasing the vessel diameter and causing new irregular bundles to appear in the zone between the sclerenchyma and late-wood xylem after 25 days and new ring vascular bundles to form in 12 weeks. These phenomena were not observed either in *R. beggeriana* “Polstjårnan” or *R. helenae* “Semiplena”, which had a high rooting percentage (Monder et al., 2019) compared with *R*. “Hurdal.” Callus overgrowth was not observed in both cultivars. Neither of the rooting enhancers under study (excluding the NAA preparation of *R*. “Semiplena”) stimulated callus proliferation (Monder et al., 2019).

In contrast to *R*. “Hurdal,” both *R*. “Polstjårnan” and “Semiplena” had a tendency for adventitious root formation along the stem, omitting its calloused basal part (Monder et al., 2019). “Hurdal” has a high tendency to an overgrowing callus, with a high percentage of cuttings developing a callus only. The use of plant origin preparations decreased the percentage of cuttings with a callus only, while the use of IBA increased it for H1 cuttings. However, none of the rooting enhancers caused an increase in the rooting ability on the internode or along the stem of *R*. “Hurdal.” The roots in “Hurdal” appeared at the base only and were obstructed by callus overgrowth. The roots of the *Rosa* “Semiplena” and “Polstjarnan” also started appearing on the basal part after the 3–4 weeks of rooting, and, later, after 6–8 weeks, they appeared in the node (“Polstjårnan”) and along the internode (“Semiplena”) (Monder et al., 2019). These phenomena were not noted for the “Hurdal” rooted cuttings, and roots were observed only at the basal part of cuttings independent of rooting enhancers.

Despite the above, the research on *R*. “Hurdal” confirmed previous observations concerning *R*. “Polstjårnan” and “Semiplena” (Monder et al., 2019). The adventitious roots omitted the sclerenchyma layer, which did not form the regular ring in any of the abovementioned roses (Monder et al., 2019). The role of sclerenchyma in difficulties in root formation has not been sufficiently proven. Pericyclic sclerenchyma can be correlated with difficulty in the rooting of some woody species (Beakbane, 1969); however, rooting capabilities are connected more with the natural ability to root easily, as in *Ficus pumila*, than with the presence of sclerenchyma tissue (Davies and Joiner, 1982).

Callus overgrowth was probably one of the main reasons for the low percentage of rooted cuttings *R*. “Hurdals.” The callus formation at the basal part of the shoot of cuttings is one of the first histological reactions occurring in response to wounding caused by severance in woody plant species (Costa et al., 2007, Amissah et al., 2008). The high percentage of cuttings with a callus only, calculated after 12 weeks, resulted in a high ratio compared with the rooting percentage in the same combination. Simultaneously, the strong growth of callus cells caused a high percentage of cuttings to develop a callus only, especially after treatments with IBA and NAA preparations and in the case of control cuttings, with a high ratio to rooted cuttings. The overgrowing callus also filled the place of the pith and broke the vascular bundles of the first ring, which were visible on cross sections after 12 weeks. The numerous callus cells may have caused compression of the first ring of wood tissue and, probably, quickly established the second ring, enabling the maintenance of wood vessel function in a changed anatomical structure. Vessels wide in diameter and wide pith rays were noted. The mean width of the pith rays for all the phenological stages of cuttings was higher for control cuttings and those treated with IBA and NAA preparations than preparations of the plant origin. In the case of H4 stage cuttings of “Hurdal,” a decrease of formation was observed in latewood. These cuttings did not root, and none of the rooting enhancers increased rooting ability.

According to previous research, the cambium has a crucial role in adventitious root formation; however, it is still not known whether new parenchyma tissues are a part of the cambium, phloem, or xylem (Fouda and Schmidt, 1994; Costa et al., 2007), also in *Rosa* “Hurdals.” Roots primordia have appeared in the cambium zone in *Rosa* “Semiplena” and “Polstjarnan” (Monder et al., 2019). Roots in woody plants can be also formed in the phloem and the pericycle zone (Haissig, 1974), as was observed in *R*. “Hurdal” 25 days after cutting and after 12 weeks of rooting.

In the present study, each of the rooting enhancers had a different influence on anatomy structure. These results are contrary to research on *Rhododendron ponticum*, where rooting powders with IBA and NAA did not affect anatomical changes in the structure of cuttings, or did so only slightly (Strzelecka, 2017). However, the results do confirm previous research on “Polstjårnan” and “Semiplena.” The application of NAA preparation caused a decrease in the wood cell diameter and the width of xylem, cambium, and phloem tissue in “Polstjårnan” cuttings as well as of the wood cell diameter and width of xylem tissue in “Semiplena.” The use of NAA, IBA, and plant origin preparations in “Semiplena” cuttings caused phloem tissue to widen (Monder et al., 2019).

The plant origin preparations positively affected the rooting percentage of *R*. “Hurdal” as well as the quality (the weight of the root system and the aboveground part) of rooted cuttings. Moreover, by the use of the aforementioned preparations, a lower percentage of cuttings with a callus only was noted. These results have certainly indicated the possibility for use of these preparations in the rooting of “Hurdal” rose cuttings and may be recommended as alternative preparations of natural origin in the ecological propagation (91/414/EEC, 2009/128/WE; OJEU), compared to the classic synthetic stimulators. Plant origin preparations could positively influence changes in the condition of cuttings and the content of bioactive components in roses (Monder and Pacholczak, 2018, 2019; Monder et al., 2020) due to supplementation with nutritive substances or unknown rooting cofactors and IAA-like substances present in the bio preparations (Nardi et al., 2015; Oosten et al., 2017). Most bio preparations of plant origin contain different substances similar to auxins in various concentrations (Crouch and Van Staden, 1993; Thorsen et al., 2010; Pacholczak et al., 2016).

## Data Availability Statement

The raw data supporting the conclusions of this article will be made available by the authors, without undue reservation.

## Author Contributions

MM designed the experiment, analyzed the data, and wrote the manuscript with contribution and revision from all the authors. MM made the growth analyses. PK and AJ performed the sample preparations and observations. AJ and MM contributed to anatomy measurements and data analyses. All authors contributed to the article and approved the submitted version.

## Funding

The experiments were supported by the National Science Center, research project No. NN 310008240.

## Conflict of Interest

The authors declare that the research was conducted in the absence of any commercial or financial relationships that could be construed as a potential conflict of interest.

## Publisher's Note

All claims expressed in this article are solely those of the authors and do not necessarily represent those of their affiliated organizations, or those of the publisher, the editors and the reviewers. Any product that may be evaluated in this article, or claim that may be made by its manufacturer, is not guaranteed or endorsed by the publisher.
